# Common position of indels that cause deviations from canonical genome organization in different measles virus strains

**DOI:** 10.1186/s12985-016-0587-2

**Published:** 2016-07-29

**Authors:** Jelena Ivancic-Jelecki, Anamarija Slovic, Maja Šantak, Goran Tešović, Dubravko Forcic

**Affiliations:** 1 University of Zagreb, Centre for research and knowledge transfer in biotechnology, Rockefellerova 10, 10 000 Zagreb, Croatia; 2 Center of Excellence for Viral Immunology and Vaccines, CERVirVac, Zagreb, Croatia; 3 Pediatric infectious diseases department, University hospital for infectious diseases “Dr. Fran Mihaljevic”, Mirogojska 8, 10 000 Zagreb, Croatia

**Keywords:** Measles virus, Genome organization, Genome editing, M-F UTR, Prolonged genome, Non-canonical strains, Indels, Mononucleotide repeats

## Abstract

**Background:**

The canonical genome organization of measles virus (MV) is characterized by total size of 15 894 nucleotides (nts) and defined length of every genomic region, both coding and non-coding. Only rarely have reports of strains possessing non-canonical genomic properties (possessing indels, with or without the change of total genome length) been published. The observed mutations are mutually compensatory in a sense that the total genome length remains polyhexameric. Although programmed and highly precise pseudo-templated nucleotide additions during transcription are inherent to polymerases of all viruses belonging to family *Paramyxoviridae*, a similar mechanism that would serve to non-randomly correct genome length, if an indel has occurred during replication, has so far not been described in the context of a complete virus genome.

**Methods:**

We compiled all complete MV genomic sequences (64 in total) available in open access sequence databases. Multiple sequence comparisons and phylogenetic analyses were performed with the aim of exploring whether non-recombinant and non-evolutionary linked measles strains that show deviations from canonical genome organization possess a common genetic characteristic.

**Results:**

In 11 MV sequences we detected deviations from canonical genome organization due to short indels located within homopolymeric stretches or next to them. In nine out of 11 identified non-canonical MV sequences, a common feature was observed: one mutation, either an insertion or a deletion, was located in a 28 nts long region in F gene 5′ untranslated region (positions 5051–5078 in genomic cDNA of canonical strains). This segment is composed of five tandemly linked homopolymeric stretches, its consensus sequence is G_6-7_C_7-8_A_6-7_G_1-3_C_5-6_. Although none of the mononucleotide repeats within this segment has fixed length, the total number of nts in canonical strains is always 28. These nine non-canonical strains, as well as the tenth (not mutated in 5051–5078 segment), can be grouped in three clusters, based on their passage histories/epidemiological data/genetic similarities. There are no indications that the 3 clusters are evolutionary linked, other than the fact that they all belong to clade D.

**Conclusions:**

A common narrow genomic region was found to be mutated in different, non-related, wild type strains suggesting that this region might have a function in non-random genome length corrections occurring during MV replication.

**Electronic supplementary material:**

The online version of this article (doi:10.1186/s12985-016-0587-2) contains supplementary material, which is available to authorized users.

## Background

Measles virus (MV) is an RNA virus with a single-stranded, negative sense, nonsegmented genome. It belongs to the genus *Morbillivirus*, family *Paramyxoviridae*. The MV genome contains six tandemly linked genes (N, P, M, F, H and L), separated by nontranscribed intergenic triplets. Genes are composed of open reading frames (ORFs) with 5′ and 3′ untranslated regions (5′ UTR and 3′ UTR, respectively). Six MV genes are flanked by a short leader transcriptional control region (TCR) at the 3′ end of the genome and a trailer TCR at its 5′ end. Although nearly 11 % of MV genome is composed of non-coding regions, the genome is arranged so that distances between ORFs are not longer than 160 nucleotides (nts). The only exception is the non-coding region between M and F genes’ ORFs (M-F UTR). Its length is 1012 nts, which is 6.4 % of the total MV genome length. M-F UTR is composed of the two by far longest untranslated regions, M gene 3′ UTR and F gene 5′ UTR, 426 and 583 nts long, respectively, and intergenic triplet (Additional file 1: Table S1). Although much investigated, the precise function of this region in MV [1–3], as well as in other *Morbilliviruses* (i.e. canine distemper virus [4, 5] and peste des petits ruminants virus [6]), is not well understood. M gene 3′ UTR and F gene 5′ UTR are not essential for MV per se, but they modulate the production of M and F proteins and influence virus replication and cytopathogenicity [3]. The suggested mechanisms include mRNA stabilization and regulation of translation [3]. Furthermore, M-F UTR is among the most variable regions in the MV genome [7–9].

As with other members of the family *Paramyxoviridae* (which now comprises solely genera formerly belonging to subfamily *Paramyxovirinae*), MV replicates efficiently only when the nucleotide length of its genome is an even multiple of 6, a requirement called the “rule of 6” [10, 11]. Each nucleoprotein (N) in the viral ribonucleoprotein complex interacts with exactly 6 nts. During copying, viral polymerase “sees” the nts in the context of N. Interaction points N1-N6 are not equivalent, as particular nts that are part of signals for polymerase can be recognized only if they are positioned in a proper N subunit point, a phenomenon called “N phase context” or “hexamer phasing” [11, 12]. With the exception of the position of the F gene start, the phase of the transcription start sites of each gene is strictly conserved between the morbilliviruses [11, 13].

The canonical MV genome organization (Additional file 1: Table S1) is characterized by its total size of 15 894 nts and precisely defined length of every genomic region [14]. We have previously described wild type measles virus strains with deviations from canonical genome organization (strains possessing insertions and deletions of one or few nts, leading to a change in the N phase context within some genomic regions, but not differing in total genome size) [8, 15]. Since 2009, measles strains with genomes extended by 6 nts in total have been detected in the USA [16] and Europe ([7], strain presented in this paper).

Like other RNA/DNA polymerases, paramyxoviral RNA-dependent RNA polymerases (RdRp) have the propensity to mistakenly insert or delete nts within homopolymeric tracts [17]. Should this happen during virus replication, it would lead to a change of total RNA length and deviation from the rule of 6. This divergence can be corrected by compensatory insertions or deletions that restore the polyhexameric length. The occurrence of such counter-mutations has been shown in a few studies. Sequence analyses of recombinant human parainfluenza virus 2 (HPIV2) [17] and HPIV3 [18] rescued from cDNAs that did not conform to the rule of 6 showed that obtained viruses contained nucleotide insertions that corrected the length of the viral genome in such a manner that it became polyhexameric. Recombinant polyploid MV containing foreign gene construct that disabled virus replication, accumulated nucleotide insertions that inactivated the foreign gene expression and possessed compensatory deletions that restored polyhexameric genome length [19].

Although programmed and highly precise pseudo-templated nucleotide additions during transcription are inherent to polymerases of all viruses belonging to the family *Paramyxoviridae*, a similar mechanism that would serve to non-randomly correct genome length has so far not been described in the context of copying a complete virus genome. During transcription, pseudo-templated nucleotide additions occur in: (a) reiterative copying of short runs (4–7 nts long) of template uridylates in polyadenylation of viral mRNAs; and (b) mRNA editing, a cotranscriptional insertion of a single non-templated G, which happens with defined frequency during P gene transcription [20–22]. During mRNA editing, polymerase stutters at the sequence 3′-UUUUUCCC-5′ on the template strand (positions 2491–2498 on genomic cDNA) and inserts an extra G, leading to a frameshift and the production of the V protein mRNA. In Sendai virus, minigenomes whose lengths did not conform to the rule of six and which contained the P gene editing site underwent in vitro nucleotide insertions or deletions within the editing site that generated polyhexameric genome lengths [20]. In a complete infectious virus, the P gene editing site is unlikely to be used for this function, as this would alter the expression of P and V proteins [22].

In order to explore whether non-recombinant measles strains showing deviations from canonical genome organization possess a common genetic characteristic, which would suggest that genome length correction is not a random process, we compiled and analysed all complete MV genomic sequences available in open-access sequence databases till 05/05/2016. During multiple sequence analyses, we identified the strains with putative indels and analysed their positions. In 9 out of 11 identified non-canonical MV sequences, a common feature was observed: one mutation, either an insertion or a deletion, was located in a 28 nts long region in F gene 5′ UTR.

## Methods

### Compilation of genomic MV sequences

Sixty-four complete genomic MV sequences were retrieved from the GenBank database (Table 1). In addition, 52 partial (nearly complete) MV sequences spanning genomic region 5051–5078 were also compiled (Additional file 2).

**Table 1:** Measles virus complete genome sequences used in sequence analyses

Strain name	Acc. no.	Genotype
Edmonston (AIK-C vaccine)^a^	AF266286	A
Edmonston (Moraten vaccine)^a, b^	AF266287	A
Edmonston (Zagreb vaccine)^a^	AF266290	A
Edmonston (Schwarz vaccine)^a, b^	AF266291	A
Edmonston Enders (Morten)^a, b^	FJ211583	A
Schwarz master seed (MEV10016)^a, b^	FJ211589	A
Schwarz lot AMJRB107B^a,^ ^b^	FJ211590	A
Schwarz FF-8^a^	AB591381	A
Edmonston wild-type strain^a^	AF266288	A
Edmonston (Rubeovax vaccine)^a^	AF266289	A
Edmonston Zagreb master seed^a,^ ^c^	AY486083	A
Edmonston Zagreb working seed^a, c^	AY486084	A
**Edmonston** ^**a**, **NC**^	K01711	A
Changchun-47^d^	EF033071	A
Changchun-47^d^	FJ416068	A
Leningrad-4	AY730614	A
Leningrad-16 master seed^e^	JF727649	A
Leningrad-16 final vaccine^e^	JF727650	A
CAM-70 vaccine lot2^f^	DQ345721	A
CAM-70 vaccine lot1^f^	DQ345722	A
CAM-70 10pCEF^f^	DQ345723	A
Shanghai-191	EU435017	A
Shanghai-191	FJ416067	A
KS	HM439386	B3
MVi/New Jersey.USA/45.05	JN635408	B3
Ichinose-B95a	NC_001498	D3
D-V/S^g^	EU293548	D3
D-CEF	EU293549	D3
Davis87^g^	EU293550	D3
D-VI	EU293551	D3
D-VII	EU293552	D3
T11wild	AB481087	D3
T11Ve-23	AB481088	D3
MVi/California.USA/8.04	JN635409	D3
**MVi/Tokyo.JPN/37.99(Y)** ^**NC**^	GQ376026	D3
**MVi/Tokyo.JPN/37.99(Y)C7** ^**NC**^	GQ376027	D3
SSPE-Kobe-1^h, NC^	AB254456	D3
SI^h^	JF791787	pending
**MVi/Treviso.ITA/03.10/1[D4]** ^**NC**^	KC164757	D4
**MVi/New York.USA/26.09/3** ^**NC**^	JN635402	D4
**MVi/Florida.USA/19.09** ^**NC**^	JN635403	D4
MVi/Washington.USA/18.08/1	JN635405	D5
MVi/Arizona.USA/11.08/2	JN635406	D5
**MVs/Zagreb.CRO/47.02/[D6] SSPE** ^**h, NC**^	DQ227318	D6
**97-45881** ^**NC**^	DQ227319	D6
**MVs/Zagreb.CRO/08.03/ SSPE** ^**h, NC**^	DQ227320	D6
**WA.USA/17.98** ^**NC**^	DQ227321	D6
MVi/California.USA/16.03	JN635410	D7
MVi/Virginia.USA/15.09	JN635404	D8
MVi/Texas.USA/4.07	JN635407	D8
MVi/Muenchen.DEU/19.13[D8]	KJ410048	D8
MVi/Venice.ITA/06.11/1[G3]	KC164758	G3
MVi/Pennsylvania.USA/20.09	JN635411	H1
IMB-1	FJ161211	H1
MVi/Zhejiang.CHN/7.05/4	DQ211902	H1
MVi/Zhejiang.CHN/10.05/1[H1]	KJ755976	H1
MVi/Zhejiang.CHN/12.09/1[H1]	KJ755980	H1
MVi/Zhejiang.CHN/10.11/2[H1]	KJ755982	H1
MVi/Zhejiang.CHN/16.10/2[H1]	KJ755981	H1
MVi/Zhejiang.CHN/12.08/1[H1]	KJ755979	H1
MVi/Zhejiang.CHN/14.07/1[H1]	KJ755978	H1
MVi/Zhejiang.CHN/12.06/2[H1]	KJ755977	H1
MVi/Zhejiang.CHN/02/2[H1]	KJ755975	H1
MVi/Zhejiang.CHN/99/2[H1]	KJ755974	H1

^a^strains belonging to Edmonston lineage

^b^identical sequences

^c^identical sequences

^d^identical sequences

^e^identical sequences

^f^identical sequences

^g^identical sequences

^h^strains isolated from patients with subacute sclerosing panencephalitis

^NC^strains showing deviations from canonical genome organization

Non-canonical strains are indicated in bold

### Preparation of viral suspensions

Isolation of MVi/Zagreb.CRO/48.03[D4] and MVi/Zagreb.CRO/19.08[D4] viruses was described in Ivancic-Jelecki et al. [23].

### RNA extraction and reverse transcription

RNA was extracted using the guanidinium isothiocyanate-phenol-chloroform method [24]. Prior to reverse transcription, RNA was denatured at 70 °C for 10 min and immediately cooled at 4 °C. Reverse transcription was performed at 42 °C for 60 min using M7 primer (5′-GGAGGAGCAGATGCAAGATA-3′) and SuperScript III reverse transcriptase (Thermo Fisher Scientific). Reaction mixture contained 3.3 pmol of primer, 1× first strand buffer (50 mM Tris-HCl (pH 8.3 at room temperature), 75 mM KCl, 3 mM MgCl_2_), 10 nmol of each dNTP, 0.25 μmol of dithiothreitol, 40 U of RNase inhibitor RNase OUT (Thermo Fisher Scientific) and 200 U of SuperScript III reverse transcriptase in a total volume of 25 μL.

### PCR amplification and sequencing

PCR amplification of M-F UTR was performed using Platinum *Pfx* DNA polymerase (Thermo Fisher Scientific) and primer pairs (a) M7 and M6 (5′-CCGTCTTGGATTGTCGATG-3′); and (b) F9 (5′-GGCCAAGGAACATACACA-3′) and F16 (5′-ATTGATGGCTGGAACGAGTC-3′). Reaction mixtures included 25 μL of cDNA (total reverse transcription mixture), 1× *Pfx* amplification buffer (Thermo Fisher Scientific), 3× PCRx Enhancer Solution (Thermo Fisher Scientific), 30 nmol of each dNTP, 0.1 μmol MgSO_4_, 30 pmol of each primer and 1 U of Platinum *Pfx* DNA polymerase in a total volume of 100 μL. After the initial denaturation step at 94 °C for 5 min, 45 cycles at 94 °C for 30 s, 50 °C for 30 s and 72 °C for 1 min were performed, followed by a terminal elongation step at 72 °C for 10 min.

Purified PCR products were sequenced on ABI PRISM 3130 Genetic Analyzer (Thermo Fisher Scientific), according to manufacturer’s instructions. Nucleotide sequences were deposited in GenBank under acc. nos. KF515521 and KF515522.

### Multiple sequence alignments, calculation of R index and visual depiction of variation

Multiple sequence alignments were performed using Clustal X v2.1, Molecular Evolutionary Genetics Analyses (MEGA) v6.06 and BioEdit v7.1.3.0 softwares.

The R index was calculated by dividing the number of mononucleotide repeats identified in an individual genomic segment with the number of nts in that segment.

For visualization of variability in 64 different complete measles genome sequences a Web-based program Fingerprint was used [25] (http://evol.mcmaster.ca/fingerprint/). In this program, the variability of a genomic position is quantified by considering the number of different residues (1–4) occurring at that position.

### MV phylogenetic analyses and genotyping

Maximum likelihood phylogenetic trees were generated using MEGA software, under the most appropriate model of nucleotide substitution determined with jModeltest v2.1.4. Bootstrap probabilities for 1 000 iterations were calculated to evaluate confidence estimates.

MV genotyping, based on the last 450 coding nucleotides of the N gene (N450), was performed according to WHO recommendations [26].

## Results

Sixty-four complete genomic MV sequences, belonging to ten different MV genotypes (out of 24), were retrieved from the GenBank database (Table 1). Some sequences were obtained after the sequencing of different samples of the same viral strain (e.g. of samples differing in passage histories). In six instances identical sequences were deposited under different names and therefore our data set contained 54 different entries.

### Measles virus strains with non-canonical genomic properties

In 11 different sequences (Table 2), deviations from canonical genome organization were identified: some regions are longer (for 1, 2 or 7 nts) or shorter (for 1 or 2 nts) due to indels.

**Table 2:** Position of putative indels in measles strains with non-canonical genome organization

Strain name (GenBank acc. no.)	Genotype	Submitted by	Insertion		Deletion		Genome length
			Mutation	Genomic region*	Mutation	Genomic region*	
WA.USA/17.98 (DQ227321)	D6	Forcic et al.	+T or + C (T_1_C_2_ or T_2_C_1_ → T_2_C_2_)	4532–4534, M gene 3′ UTR	**–N**	**5052–5078, F gene 5′ UTR**	15,894
97-45881 (DQ227319)	D6	Forcic et al.	+A (A_7_ → A_8_)	4524–4531, M gene 3′ UTR	**-N**	**5052–5078, F gene 5′ UTR**	15,894
MVs/Zagreb.CRO/47.02/[D6] SSPE (DQ227318)	D6	Forcic et al.	+A (A_7_ → A_8_)	4509–4516, M gene 3′ UTR	**–NN**	**5053–5078, F gene 5′ UTR**	15,894
			+C (C_6_ → C_7_)	4519–4525, M gene 3′ UTR			
MVs/Zagreb.CRO/08.03/SSPE (DQ227320)	D6	Forcic et al.	+A (A_6_ → A_7_)	4524–4530, M gene 3′ UTR	–A (A_4_ → A_3_)	7087–7089 F gene ORF^a^	15,894
MVi/Tokyo.JPN/37.99(Y) (GQ376026) MVi/Tokyo.JPN/37.99(Y)C7 (GQ376027) SSPE-Kobe-1 (AB254456)	D3 D3	Haga et al. Haga et al. Hotta et al.	**+N**	**5051–5079, F gene 5′ UTR**	-A (A_5_ → A_4_)	7025–7028, F gene ORF^b^	15,894
MVi/Florida.USA/19.09 (JN635402) MVi/New York.USA/26.09/3 (JN635403) MVi/Treviso.ITA/03.10/1[D4] (KC164757)	D4 D4 D4	Rota et al. Rota et al. Palù et al.	+7C (C_5_ → C_12_)	4763–4774, M gene 3′ UTR	**-A**	**5071–5076, F gene 5′ UTR**	15,900
Edmonston (K01711)	A	Cattaneo et al.	+A (A_2_ → A_3_)	29–31, leader	-A (A_6_ → A_5_)	3398–3402, P gene 3′ UTR^c^	15,894

*UTR* untranslated region, *ORF* open reading frame

Mutations within the same segment in F gene 5′ UTR (corresponding to positions within 5051–5078 region in canonical strains) are shown in bold

*nucleotide numbering corresponds to positions in genomic cDNA

^a^mutation causes frameshift after codon for amino acid 543 and translation termination after amino acid 546

^b^mutation causes frameshift after codon for amino acid 523 and translation termination after amino acid 534

^c^region used for pseudo-templated polyadenilation of P/V/C mRNAs

Epidemiologically/ancestrally/based on common genetic characteristics, 10 of these 11 strains group into three clusters:

1. WA.USA/17.98 and 97-45881 are wild type strains belonging to genotype D6. They were detected in Europe in the late 1990s [15, 27]. SSPE strains MVs/Zagreb.CRO/47.02/[D6] SSPE and MVs/Zagreb.CRO/08.03/SSPE are regionally and timely related to these two wild type strains [8, 15, 28].
2. The D3 wild type strain MVi/Tokyo.JPN/37.99(Y) was isolated in Japan in 1999 from peripheral blood mononuclear cells of a patient who died of measles-induced encephalitis. Its descendant strain MVi/Tokyo.JPN/37.99(Y)C7 was obtained after 7 passages of MVi/Tokyo.JPN/37.99(Y) on cotton rat lung cells [29]. Similar to them is a D3 SSPE virus SSPE-Kobe-1 isolated from brain tissue of a patient who contracted measles in 1999 (personal communication with Hak Hotta). The virus was isolated 6 weeks after the onset of SSPE symptoms [30].
3. Wild type strains MVi/New York.USA/26.09/3, MVi/Florida.USA/19.09 [16] and MVi/Treviso.ITA/03.10/1[D4] were isolated in Europe and America in 2009 and 2010. These 3 D4 strains are mutually highly similar, differing in 18, 23 and 27 nts from each other. Although there are no data about a possible epidemiological link among these strains, an interesting feature is that the genomes of all three of them are prolonged. M gene 3′ UTR is extended for 7 cytidines in region 4763–4744, so that a homopolymeric tract of 12 cytidine residues is created. F gene 5′ UTR is shortened for 1 nt, leading to a total genome length of 15 900 nts. We observed the same insertion and deletion in our wild type isolate MVi/Zagreb.CRO/19.08[D4].

The 11^th^ strain in which mutations were observed is a strain belonging to the Edmonston lineage, submitted to GenBank under the name Edmonston, acc. no. K01711 [31]. In addition to this strain, 12 other sequences included in our analysis belong to the Edmonston lineage. They represent various vaccine strains (or different seeds of a same vaccine) that have all originated from a single wild type isolate [32]. In none of these 12 remaining Edmonston sequences were deviations from canonical genome organization observed.

### Genomic positions of indels

The positions of identified indels are presented in Table 2. Mutations occurred either in polyadenosine, polyguanosine or polycytidine stretches or in positions next to them (e.g. the position of insertion in strain WA.USA/17.98 is located immediately after 7 nts long polyadenosine stretch). In all strains compensatory mutations were identified and the rule of 6 was conformed to. In SSPE strain MVs/Zagreb.CRO/47.02/[D6] SSPE two sites of insertions of a nucleotide were identified. Deletion of two nts was detected in a single downstream region.

With the exception of Edmonston, in all strains insertions are in M-F UTR and deletions are either in F gene 5′ UTR or in F gene ORF. Deletions in F gene ORF caused frameshifts and led to truncations of the cytoplasmic tail of F protein’s F1 subunit, a feature often found in SSPE strains. Besides the two SSPE strains MVs/Zagreb.CRO/47.02/[D6] SSPE and SSPE-Kobe-1, a deletion in F gene ORF was also detected in MVi/Tokyo.JPN/37.99(Y) and MVi/Tokyo.JPN/37.99(Y)C7, viruses that descended from a wild type strain that had caused a lethal encephalitis.

Excluding MVs/Zagreb.CRO/08.03/SSPE and Edmonston, in all strains one of the indels is placed within the 28 nts long segment in F gene 5′ UTR, located at positions 5051–5078 in the genomic cDNA of canonical strains (shown in bold in Table 2). The only non-canonical strain in which one deviation is placed before and the other after 5051–5078 segment (i.e. RdRp did not insert compensatory mutation in this region during genome/antigenome copying) is the SSPE strain MVs/Zagreb.CRO/08.03/SSPE.

The specificities found in the Edmonston sequence were not detected in any other of analysed strains. It is the only sequence where the insertion site is located in the leader region and the deletion site is placed in a region used for P mRNA polyadenylation. The insertion of an A in the leader sequence disrupts the highly conserved replication promoter element positioned within the N gene [22]. For morbilliviruses, this element has the sequence 3′-(C^1^n^2^n^3^n^4^n^5^n^6^)_3_-5′ (numbers in superscript indicate N phase context; the element’s position corresponds to region 79–96 in genomic cDNA, nts 79, 85 and 91 being Gs) [22]. The nucleotide at position 85 in the Edmonston genomic cDNA sequence is A. Furthermore, the insertion located in the leader region leads to a change of the N phase contexts of transcription start signals of the N and P genes and of the transcription stop signal of the N gene. The phasing of the mRNA editing site is also changed. None of these sites are found in a random N phase context within morbilliviruses [11].

### Indels in 5051–5078 segment

**Fig. 1:**
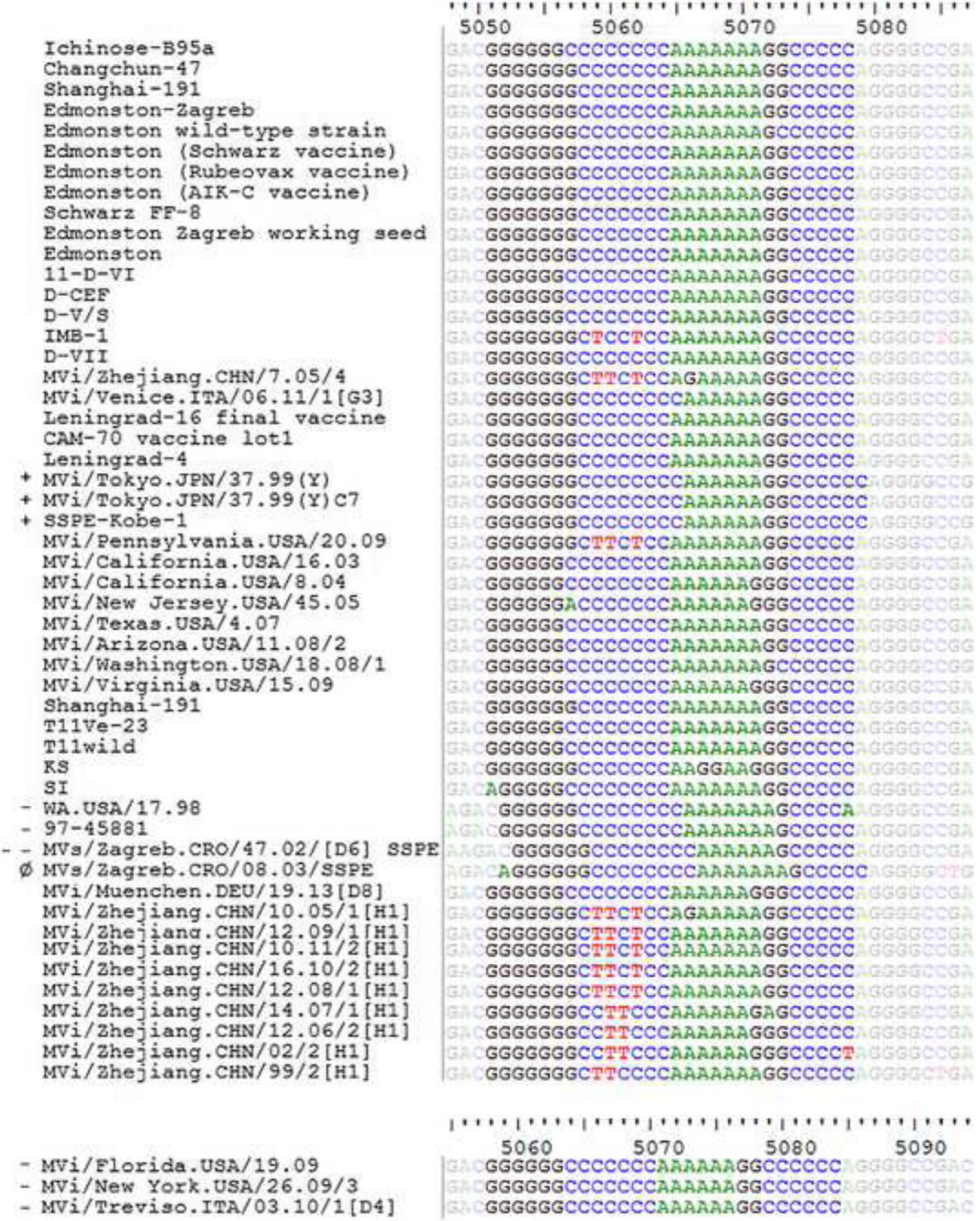
Multiple sequence alignment of measles genomic cDNA, showing a segment of F gene 5′ untranslated region. Legend: Nucleotides at positions 5051–5078 (or at corresponding positions in non-canonical strains) are highlighted. Strains in which insertions or deletions were detected in 5051–5078 region are indicated with plus or minus, respectively. A strain in which the insertion is located before and the deletion after 5051–5078 segment is indicated with Ø

The consensus sequence of the 5051–5078 segment in canonical strains is G_6-7_C_7-8_A_6-7_G_1-3_C_5-6_, the total number always being 28. The sequence of this region in 54 different MV strains is presented in Fig. 1. Non-canonical strains, with insertions or deletions in this segment, are indicated by the plus and minus sign, respectively.

We searched through partial MV entries in the GenBank database in order to find additional sequences of the 5051–5078 segment. Fifty-two sequences were retrieved, plus the two that we sequenced during the course of this study (wild type isolates MVi/Zagreb.CRO/48.03[D4] and MVi/Zagreb.CRO/19.08[D4]). The sequences were from strains belonging to the B3 (11 strains), D4 (7 strains), D8 (35 strains) or H1 (1 strain) genotypes. Indels were identified only in D4 strains, in all of them except in the oldest one, MVi/Zagreb.CRO/48.03[D4] (oldest not only by chronology of detection of D4 strains included in this study, but also by its position on the phylogenetic tree (Additional file 3: Figure S1). Position of indels are identical as in strains MVi/New York.USA/26.09/3, MVi/Florida.USA/19.09 and MVi/Treviso.ITA/03.10/1[D4].

### Distribution of homopolymeric sequences in measles genomes

All mutations identified during our study occurred either in homopolymeric stretches or in positions next to them. In order to investigate the locations and distribution pattern of mononucleotide repeats in MV strains, we identified all positions where minimally 5 nts long mononucleotide repeats are present. Analysis included all 54 different complete genomic sequences and only repeats found in at least two non-temporally and non-geographically related strains were counted.

**Fig. 2:**
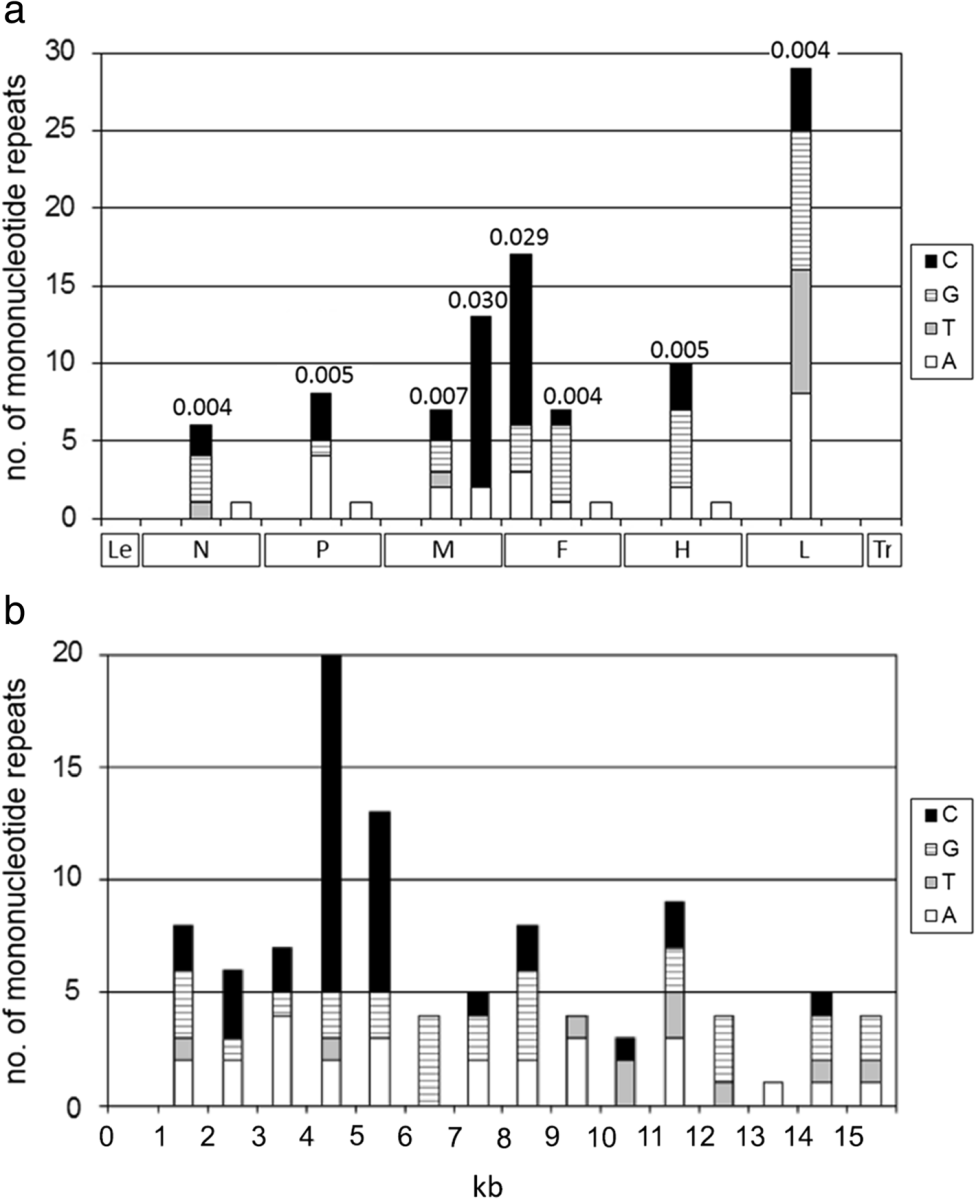
Number of mononucleotide repeats (of length ≥5 nucleotides) present in measles strains. Legend: **a** Measles virus cDNAs on x-axis is divided into leader region (Le), individual genes and trailer region (Tr); each gene is divided into 5′ untranslated region (UTR), open reading frame and 3′ UTR, separated by ticks on the x-axis. Values above bars indicate the number of repeats relative to segment length. **b** Measles virus cDNAs on the x-axis is divided into 1 kilobase-long segments

The total number of homopolymeric runs was 37, 28, 26 and 10 for polycytosines, polyguanosines, polyadenosines and polythymidines, respectively. The distribution of repeats is shown in Fig. 2. With the exception of M-F UTR, the only mononucleotide repeats found in non-coding regions are the ones used for pseudo-templated polyadenylation of mRNAs (Fig. 2a). Homopolymeric runs were identified throughout the entire genome length except in the first 1 000 nts (numbering corresponding to genomic cDNA) (Fig. 2b). Considering that individual genomic segments (i.e. the coding and two non-coding regions of each gene) have different lengths, we calculated the R index, which indicates the number of repeats relative to segment length. While the coding regions have an R index in the range of 0.004–0.007, the R index of M gene 3′ UTR and F gene 5′ UTR is 0.030 and 0.029, respectively. These segments are especially rich in polycytosine repeats (viewed in genomic cDNA; Fig. 2a).

Although quite a large number of homopolymeric runs were identified in M gene 3′ UTR and F gene 5′ UTR, 13 and 17 respectively, indels were found in no more than 9 of them. This indicates that not all parts of this long non-coding region can tolerate such mutations, despite the fact that it is among most variable parts of the genome (Additional file 4: Figure S2, [7–9]).

The 12-cytosine homopolymer detected in F gene 5′ UTR in MVi/New York.USA/26.09/3, MVi/Florida.USA/19.09, MVi/Treviso.ITA/03.10/1[D4] and MVi/Zagreb.CRO/19.08[D4] (strains with prolonged genome), created by the insertion of an additional 7 cytosines into a 5-cytosine stretch, is the longest mononucleotide repeat identified in any of the analysed strains.

## Discussion

The complete genomic organization of MV was deduced in the late 1980s [33]. Unlike some other virus species belonging to the *Paramyxoviridae* family, which are known to possess few different genomic lengths (e.g. Newcastle disease virus, as well as other avian paramyxoviruses within the genus *Avulavirus* [34]), MV genomic length and organization was for a long time considered to be uniform [14].

Until 2012 (when sequences of MV strains with prolonged genomes were released) and the publication of Bankamp et al., which describes these viruses [16], only rarely were reports of strains possessing non-canonical genomic properties published [8, 15], and even in those reports observed indels were mentioned only marginally.

Eleven complete genomic sequences with non-canonical properties analysed in this paper were submitted to open public databases by six different research groups (including ours), making it less likely that their specificities resulted from errors in RT-PCR or in sequencing. Ten of the 11 strains are grouped in three clusters. There are no indications that these clusters are somehow evolutionary linked, other than the fact that they all belong to clade D.

The 11^th^ non-canonical sequence was obtained from a sample containing the Edmonston strain. A suggestion that mistakes might have occurred during the sequencing of this sample, which was done in the 1980s and early 1990s, was made by Bankamp et al. [16] although sequence submitters claim otherwise (personal communication with M. Billeter). As this virus was extensively passaged in vitro, it is possible that this has led to the origin of the infectious Edmonston-lineage virus possessing such genomic sequence.

In nine non-canonical strains (all except Edmonston and MVs/Zagreb.CRO/08.03/SSPE), one of the genome editing sites is located within a 28 nts long segment in F gene 5′ UTR, which is composed of five tandemly linked homopolymeric stretches. None of these five stretches has a definite length in canonical strains. The mutations detected in this region include both insertions and deletions. Compensatory mutations (leading to the re-establishment of polyhexameric length) were located in adjacent regions, M gene 3′ UTR and F gene ORF, so that the N phase contexts of start and stop signals of downstream genes were not changed. During the preparation of this manuscript, we sequenced M-F UTR of a D8 wild type strain that circulated in Croatia in 2014–2015 (GenBank acc. nos. KX555602 and KX555601 for N450 and M-F UTR, respectively) and found that it also possess an insertion of a nucleotide in 5051–5078 segment. Accompanying deletion is at nucleotide position 4714 or 4715, in M gene’s 3′ UTR (data not shown).

As discussed by Skiadopoulos et al. [17], the genome length correcting mechanism could operate by involving either (a) random length corrections, followed by a stringent selection for virus in which the correction was close to the point of deviation, or (b) non-random length corrections, involving a replication complex that “senses” the deviation from the rule of 6 and acts to insert a correcting mutation at a second, downstream site in the nascent molecule. Our analysis favours the second hypothesis, as the same narrow genomic region was found to be mutated in different, non-related measles strains.

Indels detected in the sequence of Edmonston and MVs/Zagreb.CRO/08.03/SSPE show that also other mechanisms can be involved in genome length corrections. A similar result was obtained with recombinant MVs: Rager et al. [19] found that recombinant MV, with a foreign gene fused to H gene’s C terminus, disabled the expression of the foreign gene due to the insertion of an A in an A_6_G_4_ region. Compensating deletion occurred downstream, in the L gene coding region where an A was deleted from an A_5_G_4_ sequence. Other clones carried an A deletion in a G_2_A_5_ region of the foreign gene and the polyhexameric length was restored by the insertion of an A in different polyadenylation sites. None of the sites reported by Rager et al. [19] to be involved in genome length corrections were located within the 5051–5078 region.

Generally, studies that investigated genome length corrections of viruses belonging to the family *Paramyxoviridae* [17–20] reported that inserted or deleted residues were adenosines, uridines or guanosines. We found that cytidines can also be inserted, but this may be a consequence of insertion occurring during the synthesis of antigenomic RNA. Skiadopoulos et al. [17] proposed a hypothesis that the fact that they found only adenosines and uridines to be inserted or deleted might simply reflect a lower content of homopolymeric guanosines and cytidines in the regions most amenable to accepting a length correction, namely the non-translated regions and intergenic regions. With the exception of M gene 3′ UTR and F gene 5′ UTR, MV non-coding regions are relatively short and do not contain homopolymers other than polyuridylates used for polyadenylation of viral mRNAs. In contrast, M gene 3′ UTR and F gene 5′ UTR are the regions with the largest numbers of homopolymers relative to their length. Even when the absolute numbers of homopolymers are compared, the only region with more mononucleotide repeats is the 6.5 kb long L gene ORF. Therefore, it is not surprising that nearly all of identified indels were in M-F UTR.

Mononucleotide repeats are generally considered to be exceptionally unstable genetic elements, prone to indels [35]. In most bacterial genes they are underrepresented in coding regions [36, 37], as they lead to high error rates of transcription [38] and translation [39]. The finding that 9 out of 10 wild-type non-canonical strains possess an indel within the same 28 nts long region was rather unexpected, as 26 other homopolymeric runs (of length ≥5 nts) were identified in M-F UTR, outside the 5051–5078 segment.

Presumably, MV has maintained a significant non-coding nucleotide sequence content for its functionally important regulatory elements. Known MV regulatory sequences (summarized in Parks et al. [40]) located within non-coding regions are promotor sequences, TCRs at genomic ends, gene end and gene start sequences, as well as intergenic regions that guide transcription termination and reinitiation. A specific regulatory function of F gene’s 5′ UTR is its involvement in the determination of AUG that is used as the F protein start codon [2].

Since the compact genomic organization and high-coding capacity of genes offer a selective advantage for rapidly replicating RNA viruses [41], long, highly variable M-F UTR is likely to be present and evolutionary preserved because of its functionally important (and yet unknown) regions.

## Conclusions

A common narrow genomic region that harbours an indel mutation in 9 out of 11 of so far completely sequenced non-canonical measles strains was identified (segment 5051–5078 in canonical strains). The fact that it was found to be mutated in different, non-related, wild type strains suggests that this region might have a function in non-random genome length corrections occurring during MV replication.

## Abbreviations

HPIV, human parainfluenza virus; M-F UTR, non-coding region between M and F genes’ ORFs; MV, measles virus; N, nucleoprotein; N450, the last 450 coding nucleotides of the N gene; nts, nucleotides; ORF, open reading frame; RdRp, RNA-dependent RNA polymerases; TCR, transcriptional control region; UTR, untranslated region

## Additional files

Additional file 1: Table S1. Length of individual genomic regions in measles strains with canonical genome organization. (DOC 49 kb) Additional file 2: List of partial measles virus sequences that include genomic region 5051–5078. (TXT 827 kb) Additional file 3: Figure S1. Phylogenetic tree of D4 measles strains, constructed using Edmonston-Zagreb as outgroup. Legend: A) Phylogenetic tree based on N450 genomic segment (standard measles virus genomic region used in genotyping). It was generated using the maximum-likelihood method, under the Kimura-2 parameter model. B) Phylogenetic tree based on untranslated region between M and F genes’ ORF (1012 and 1018 nucleotides for canonical and non-canonical strains, respectively; the most variable part of the measles virus genome). It was generated using the maximum-likelihood method, under the Tamura-Nei model (TN93). Scale bars indicate the proportion of nucleotide substitutions. Numbers are bootstrap values determined for 1000 iterations. (TIF 1253 kb) Additional file 4: Figure S2. Variability fingerprint constructed using 54 different complete measles genomic sequences. Legend: The variability of each genomic position is quantified by considering the number of different nucleotides that are found at that position (shown in white, light grey, dark grey or black). The rectangle indicates which fingerprint area depicts variability of non-coding region between M and F genes’ open reading frames (1012 nts). (TIF 501 kb)
