# Supplementary material for: Common position of indels that cause deviations from canonical genome organization in different measles virus strains
Source: Virol J. 2016 Jul 29;13:134. doi: 10.1186/s12985-016-0587-2 (PMC4966754; doi:10.1186/s12985-016-0587-2)
Supplement: Additional file 1: Table S1. — Length of individual genomic regions in measles strains with canonical genome organization. (DOC 49 kb) [file 12985_2016_587_MOESM1_ESM.doc]

Table S1

Length of individual genomic regions in measles strains with canonical genome organization

| **Genomic region** | **Length** | **Position**^a^ |
| --- | --- | --- |
| leader | 52 | 1-52 |
| IG | 3 | 53-55 |
| N gene |  |  |
| 5’ UTR | 52 | 56-107 |
| ORF | 1578 | 108-1685 |
| 3’ UTR | 59 | 1686-1744 |
| IG | 3 | 1745-1747 |
| P gene |  |  |
| 5’ UTR | 59 | 1748-1806^b^ |
| ORF-P | 1524 | 1807-3330 |
| ORF-V^c^ | 899 | 1807-2705 |
| ORF-C | 561 | 1829-2389 |
| 3’ UTR | 72 | 3331-3402^b^ |
| IG | 3 | 3403-3405 |
| M gene |  |  |
| 5’ UTR | 32 | 3406-3437 |
| ORF | 1008 | 3438-4445 |
| 3’ UTR | 426 | 4446-4871 |
| IG | 3 | 4872-4874 |
| F gene |  |  |
| 5’ UTR | 583 | 4875-5457 |
| ORF | 1653 | 5458-7110 |
| 3’ UTR | 137 | 7111-7247 |
| IG | 3 | 7248-7250 |
| H gene |  |  |
| 5’ UTR | 20 | 7251-7270 |
| ORF | 1854 | 7271-9124 |
| 3’ UTR | 84 | 9125-9208 |
| IG | 3 | 9209-9211 |
| L gene |  |  |
| 5’ UTR | 22 | 9212-9233 |
| ORF | 6552 | 9234-15785 |
| 3’ UTR | 69 | 15786-15854 |
| IG | 3 | 15855-15857 |
| trailer | 37 | 15858-15894 |

IG, intergenic region

UTR, untranslated region

ORF, open reading frame

^a^nucleotides are numbered 1-15894, corresponding to positions in genomic cDNA.

^b^length and positions of untranslated regions in the P gene are shown relative to open reading frame coding for P protein (ORF-P).

^c^V protein mRNA is obtained after co-transcriptional addition of a single G in cDNA region 2496-2498
