# Supplementary figures and images for: Common position of indels that cause deviations from canonical genome organization in different measles virus strains
Source: Virol J. 2016 Jul 29;13:134. doi: 10.1186/s12985-016-0587-2 (PMC4966754; doi:10.1186/s12985-016-0587-2)

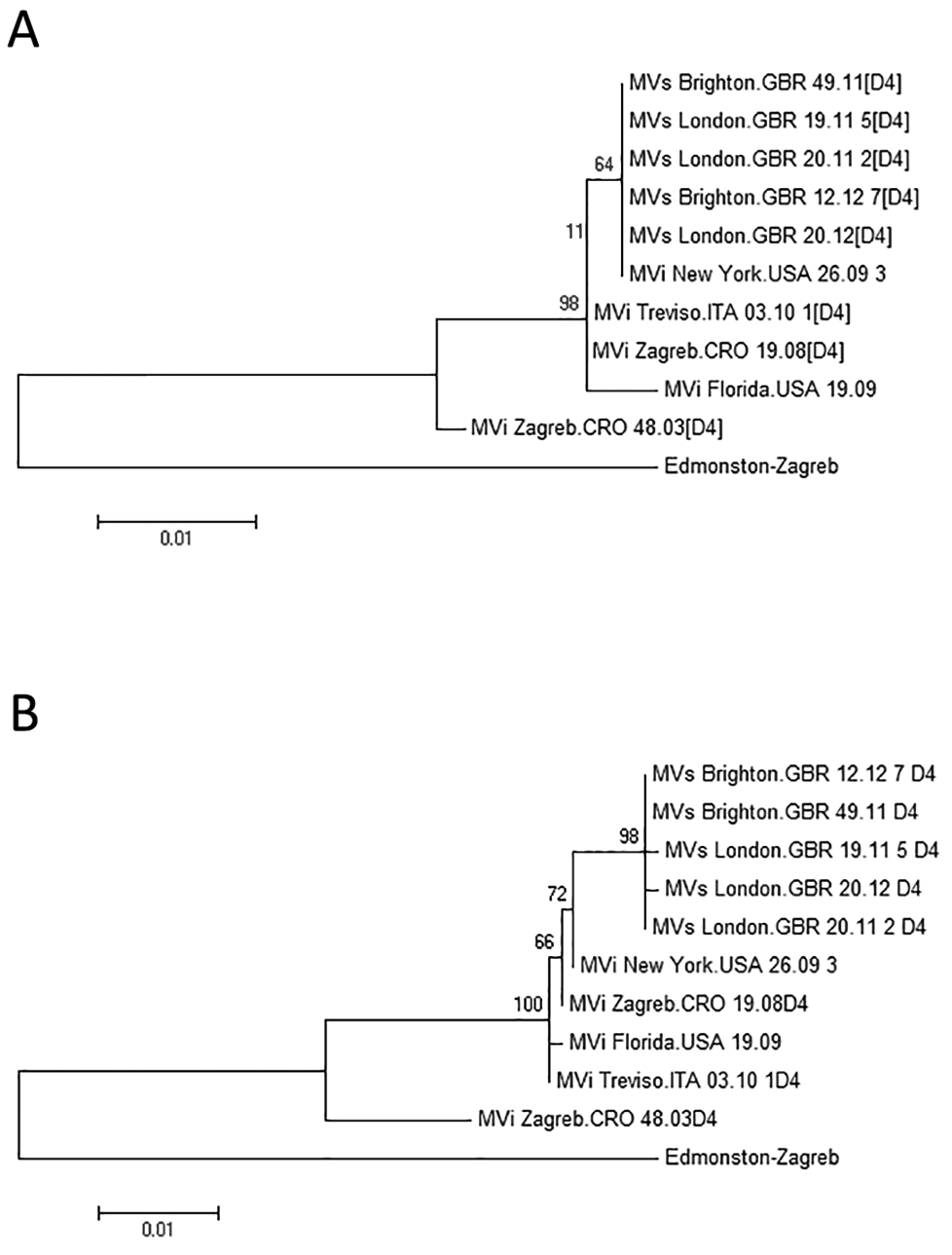

Supplement: Additional file 3: Figure S1. — Phylogenetic tree of D4 measles strains, constructed using Edmonston-Zagreb as outgroup. Legend: A) Phylogenetic tree based on N450 genomic segment (standard measles virus genomic region used in genotyping). It was generated using the maximum-likelihood method, under the Kimura-2 parameter model. B) Phylogenetic tree based on untranslated region between M and F genes’ ORF (1012 and 1018 nucleotides for canonical and non-canonical strains, respectively; the most variable part of the measles virus genome). It was generated using the maximum-likelihood method, under the Tamura-Nei model (TN93). Scale bars indicate the proportion of nucleotide substitutions. Numbers are bootstrap values determined for 1000 iterations. (TIF 1253 kb) [file 12985_2016_587_MOESM3_ESM.tif]
